# Equivalent Circuit Modeling for a Valveless Piezoelectric Pump

**DOI:** 10.3390/s18092881

**Published:** 2018-08-31

**Authors:** Jianhui Zhang, Yuan Wang, Jun Huang

**Affiliations:** 1 College of Mechanical and Electrical Engineering, Guangzhou University, Guangzhou 510006, China; 2 College of Communication Engineering, Army Engineering University of PLA, Nanjing 210007, China; linyuanju4455@163.com; 3 National Research Center of Pumps, Jiangsu University, Zhenjiang 212013, China

**Keywords:** piezoelectric, pump, valveless, treelike bifurcate, equivalent circuit

## Abstract

Various kinds of the models had been proposed to explain the relationship between the performance and the structural parameters of valveless piezoelectric pumps, so as to evaluate the functional performance such devices. Among the models, the equivalent circuit model, which converts the multi-field problem of a valveless piezoelectric pump system into a simple circuit problem, is the most simple and clear one. Therefore, the proposed structure and working principle of the valveless piezoelectric pump with multistage Y-shape treelike bifurcate tubes are analyzed; then, the equivalent circuit model of the valveless piezoelectric pump is established based on the working principles of this pump and liquid-electric analogy theory. Finally, an experimental study of the pump is carried out, with a comparative analysis of the experimental results and the simulation results of the generated equivalent circuit. The experimental results show that with a driving voltage of 100 V and frequency of 6 Hz, the maximum flow rate of the valveless piezoelectric pump is 1.16 mL/min. Meanwhile, the output current of equivalent circuit also reaches its peak at the frequency of 6 Hz, therefore, indicating a good predictive ability of this model in calculating the maximum output flow rate and best working frequency of valveless piezoelectric pumps.

## 1. Introduction

With the technical development of MEMS, various sensors and actuators, such as piezoelectric micropumps, ultrasonic motors and piezoelectric energy harvesters, have been invented and successfully applied in various kinds of micro-systems [1,2,3,4,5,6,7,8,9,10,11,12,13]. Valveless piezoelectric pumps, as a kind of micropump without moving parts inside, have drawn widespread attention due to their simple structure and absence of electromagnetic interference. Moreover, their functional integration has been continuously developed, especially in the research and application of piezoelectric pump water-cooling systems [14,15,16,17,18,19]. 

Among the various types of the valveless piezoelectric pumps, the valveless piezoelectric pumps with Y-shaped tubes, which have a simple and unique bifurcation structure, have been widely studied [20,21,22]. The natural treelike bifurcation system, due to its small drag and ideal dimensions, which can be used for minimizing the energy loss, has been designed into microstructures for energy transmission systems and thermal conversion systems. Therefore, based on Y-shape tubes, the tubes with treelike bifurcation structure were constructed for application in the microfluidic transmission and heat conduction fields. 

With the in-depth ongoing research of valveless piezoelectric pumps domestically and abroad, various kinds of the models had been proposed to explain the relationship between the performance and the structural parameters of valveless piezoelectric pumps, so as to evaluate the functional performance of the devices. Ullmann and Stemme et al. [23,24] proposed a dynamic model for predicting the maximum flow rate of a micro-pump based on the known volume changes caused by the movement of a piezoelectric vibrator. Then Olsson [25] and Ullmann [26] modified the dynamic model, taking into consideration the inertia effect of the acceleration speed of the fluid in the tubes. However, this model was incapable of describing the dynamic changes inside the piezoelectric pump. Therefore, Ullmann et al. [27] proposed an improved dynamic model to determine the dynamic behavior of the piezoelectric pump. Since the changes of fluid flow and pressure in the chamber caused by the vibration of piezoelectric vibrator were not correctly considered in these studies, Azarbadegan [28] developed a fluid-solid coupling model to evaluate the response characteristics of the valveless piezoelectric pump under resonance. Singh et al. [29] presented analytical modeling of a planar valveless micropump to predict the natural frequency and flow rate performance of the proposed micropump.

However, the abovementioned models are solid in theory but complicated in calculation; moreover, as a result of multi-physics field coupling problems such as mechanical-electric coupling and fluid-solid coupling, these models must be simplified in adopting mathematical models to analyze practical problems, resulting in errors. To decrease the complexity of the analytical model and reduce the calculation time, based on liquid-electric analogy theory, Morganti [30], Bourouina [31] and Hsu et al. [32,33] developed the equivalent circuit model of valveless piezoelectric pump with tapered tubes, and translated the multi-field problem of valveless piezoelectric pump systems into a circuit problem for solution. Therefore, in this study, the equivalent circuit model of the valveless piezoelectric pump with multistage Y-shape treelike bifurcate tubes is also proposed based on liquid-electric analogy theory to demonstrate its effectiveness, so as to lay a foundation for the optimization and improvement of the valveless piezoelectric pump.

## 2. Construction of the Device

**Figure 1:**
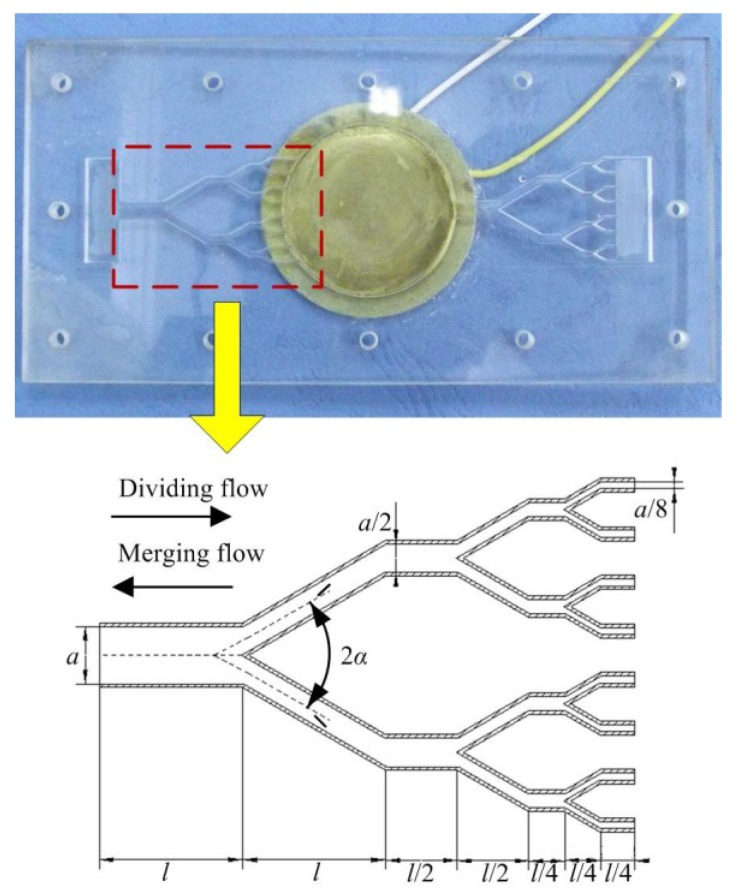
Valveless piezoelectric pump with multistage Y-shape treelike bifurcate tubes.

Based on the fractal model of biological vascular network [34], our research group has designed the multistage Y-shape tube in 2013, and successfully developed a valveless piezoelectric pump with multistage Y-shape treelike bifurcate tubes [35,36]. A sketch of the multistage Y-shape treelike bifurcate tube and a photograph of the piezoelectric pump are shown in Figure 1.

The width of mother tube of the whole tube is defined as *a,* and the outlet widths of daughter tubes of each level is half of that of the upper level. The length of mother tube is defined as *l*, and the lengths of daughter tubes of each level is half of that of upper level (except for the level I sub-tubes and the end level sub-tubes); the bifurcation angle of the tube is defined as 2*α*, and the depth of the whole tube is defined as *h*. The geometric parameters and material properties of tubes and piezoelectric vibrator are shown in Table 1. 

Taking the tube as the valve body of valveless piezoelectric pump that is without moving parts, the valveless piezoelectric pump with multistage Y-shape treelike bifurcate tubes consists of a piezoelectric vibrator, pump chamber and a pair of multistage Y-shape treelike bifurcate tubes. When the fluid flows in the tube, the energy consumption is different if different outlets are taken, namely taking the 2*i* (*i* = 1, 2, 3 …) daughter tubes as outlet (defined as dividing flow) and the mother tube as outlet (defined as merging flow), it means there are flow resistance differences between dividing flow and merging flow.

The piezoelectric vibrator will perform a vertical reciprocating motion when loaded with an alternating voltage, resulting in periodic volume changes of the pump chamber and hence the movement of fluid in the chamber. When the vibrator uplifts outward, the volume of the pump chamber increases, and the fluid flows into the chamber through the Y-shape treelike bifurcate tubes; when the vibrator depresses inward, the volume of chamber decreases, and the fluid flows out of the chamber through the tubes. Due to the different flow resistance of the merging flow and dividing flow of the fluid in the Y-shaped treelike bifurcate tube, the tube now works as a valve, resulting in different flow rates between chamber inflow and outflow from both the Y-shape treelike bifurcate tubes during a vibration period. With the continual motion of the piezoelectric vibrator, the fluid in the pump chamber macroscopically shows that it flows from inlet to outlet, resulting in a flow in a single direction.

## 3. Experimental Setup

**Figure 2:**
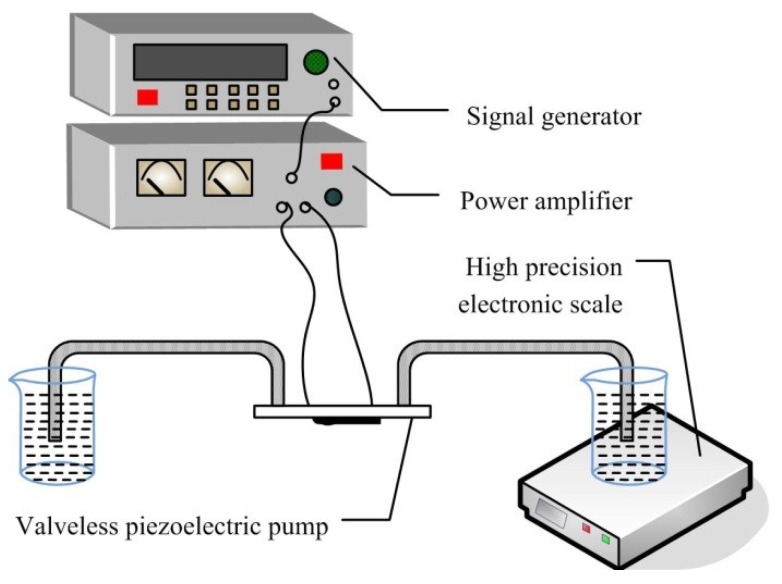
Schematic diagram of the flow experiments.

Figure 2 is a diagram of the flow experiments. For the pumping flow test, a voltage of 100 V is used to drive the piezoelectric vibrator, and deionized water is utilized as working fluid. By changing the driving frequency, the output mass flow of the piezoelectric pump in a unit time is measured, thereby the test values of flow that changes with frequency under voltage of 100 V are obtained for the piezoelectric pump. 

## 4. Modeling

### 4.1. Definition of Basic Parameters

According to liquid-electric analogy theory, when a fluid flows through a fluidic element, the pressure drop ∆*p* across the element is defined as the voltage *U*, and the flow volume *Q*_V_ passing through the element can be treated as current *I*. Therefore, the conception of fluid resistance, fluid inductance and fluid capacitance are defined as follows [37]. 

#### 4.1.1. Fluid Resistance 

When fluid is flowing in the tubes, it shows a resistance due to the viscosity force, resulting in an energy loss, shown as a pressure drop. Therefore, like the expression of electric resistance, the fluid resistance of a certain fluid part in steady flow is defined as the ratio between the pressure drop at both ends and the flow volume passing through it, which is:

(1) $$R=\Delta p/Q_{V}$$

Because the vibration of the piezoelectric vibrator in a valveless piezoelectric pump is small in scale, it can be assumed that the flow in the pump is a laminar flow. According to Poiseuille’s law, the relationship between pressure drop and flow rate is as follows:

(2) $$\Delta p=\frac{32\mu lv}{D^{2}}$$

where *v* is the flow rate of the fluid, and the flow volume is:

(3) $$Q_{V}=\frac{\pi D^{2}v}{4}$$

Therefore, the fluid resistance of tube in laminar flow is:

(4) $$R=\frac{128\mu l}{\pi D^{4}}$$

where *μ* is the viscosity coefficient, *l* is the length of the tube and *D* is diameter of the tube. 

#### 4.1.2. Fluid Capacitance 

Fluid is compressible, which can be shown under large pressure changes for liquids. In terms of the container, the internal fluid body would have the capacitive damping against the compressive deformation. According to the definition of electric capacitance, the fluid capacitance can be defined as the ratio of changes of flow volume and changes of pressure that results in its deformation, namely:

(5) $$C=\frac{\int Q_{V}dt}{\Delta p}$$

The compressibility of fluid can be described by the bulk modulus, namely:

(6) $$\kappa =-\frac{d(\Delta p)}{dV/V}$$

where *V* is the volume of the fluid. Therefore, the fluid capacitance of the tube can be represented as:

(7) $$C=\frac{V}{\kappa }$$

#### 4.1.3. Fluid Inductance 

In the fluid transmission unit, high speed transient flow occurs for the flow field inside. The fluid matter is accelerated or decelerated due to fluid inertia, resulting in a pressure variation. Therefore, the expression of fluid inductance is represented as the ratio of pressure changes aroused by both ends of fluid part and the change rate of flow volume, namely:

(8) $$L=\frac{\Delta p}{dQ_{V}/dt}$$

According to [37], this expression can be transformed into:

(9) $$L=\frac{\rho l}{A}$$

### 4.2. Equivalent Circuit of Piezoelectric Vibrator

As a structural unit, the piezoelectric vibrator will have elastic deformation when an alternating current is applied, resulting in an elastic effect and an inertial effect. Therefore, electrical inductance and capacitance effects are introduced. Meanwhile, the fluid motion in pump chamber caused by the vibration of the vibrator can be treated as a source of voltage. According to [30], the expression of electric capacitance of piezoelectric vibrator can be described as follows:

(10) $$C_{v}=\frac{{\left(\gamma A\right)}^{2}}{k}$$

(11) $$k=\frac{64}{12(1-\upsilon^{2})}\frac{\gamma \pi Eh^{3}}{{\left(D/2\right)}^{2}}$$

where *k* is the equivalent spring constant; *E* is elastic modulus of the vibrator; *ν* is the Poisson ratio of vibrator; *h* is the thickness of the vibrator; *γ* is the equivalent coefficient, which is related to the loading method and boundary conditions of the vibrator. 

Hence, the expression electrical inductance of piezoelectric vibrator can be described as follows:

(12) $$L_{v}=\frac{m_{\mathrm{eff}}}{{\left(\gamma A\right)}^{2}}$$

(13) $$m_{\mathrm{eff}}=\gamma (m_{\mathrm{membrane}}+m_{\mathrm{actuator}})$$

where *m*_eff_ is the equivalent mass of the vibrator; *m*_membrane_ is the mass of the base; and *m*_actuator_ is the mass of the piezoelectric ceramics. 

**Figure 3:**
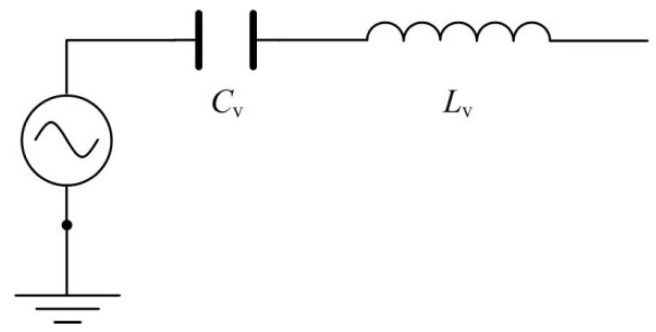
Equivalent circuit of a piezoelectric vibrator.

From the analysis above, the equivalent circuit of a piezoelectric vibrator can be obtained, as shown in Figure 3. 

### 4.3. Equivalent Circuit of the Fluid Domain 

The components of the valveless piezoelectric pump with multistage Y-shape treelike bifurcate tubes are analyzed based on the basic parameters defined by liquid-electrical analogy method, so as to obtain the equivalent circuit of the valveless piezoelectric pump. 

Since the cross-sectional area of the pump chamber is far larger than that of the tubes, only the fluid capacitance and flow tube effects are considered in the liquid-electrical equivalent of that part, while the influence of fluid resistance is ignored. Therefore, the fluid capacitance and fluid inductance of the pump chamber are defined as follows:

(14) $$C_{c}=\frac{A_{c}h_{c}}{K}$$

where $A_{c}$ and $h_{c}$ are the cross-sectional area and depth of the pump chamber:

(15) $$L_{c}=\frac{\rho h_{c}}{A_{c}}$$

**Figure 4:**
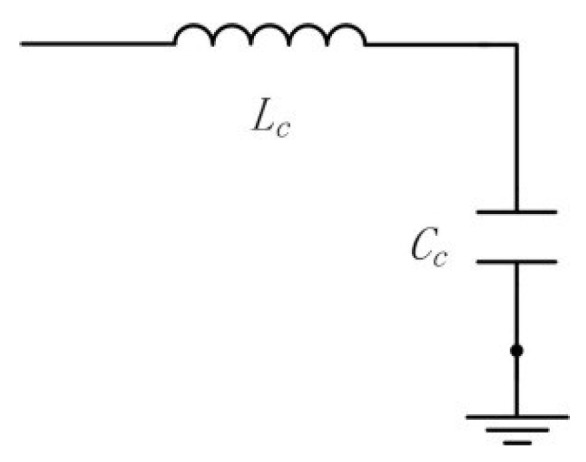
Equivalent circuit of the pump chamber.

Hence, the equivalent circuit of that part is shown as in Figure 4. 

**Figure 5:**
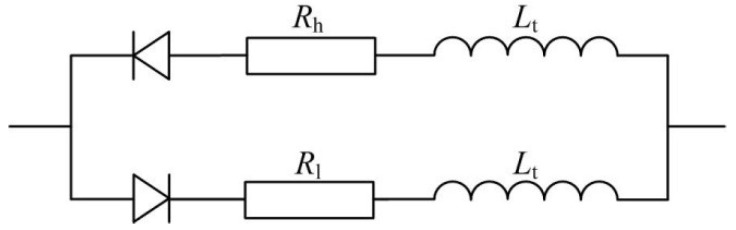
Equivalent circuit of tubes with fluid resistance differences.

Different from moving part valves, tapered flow tubes, Tesla tubes and Y-shaped tubes and other flow resistance differential type tubes have no switch actions when piezoelectric pumps are working, therefore, the fluid capacitive effect is ignored and only the influences of fluid inductance and fluid resistance are considered. At the same time, due to different loss coefficients along the positive and negative directions of the fluid in these types of tubes, different fluid resistances are shown. Therefore, the equivalent circuit of this part is as shown in Figure 5. 

**Figure 6:**
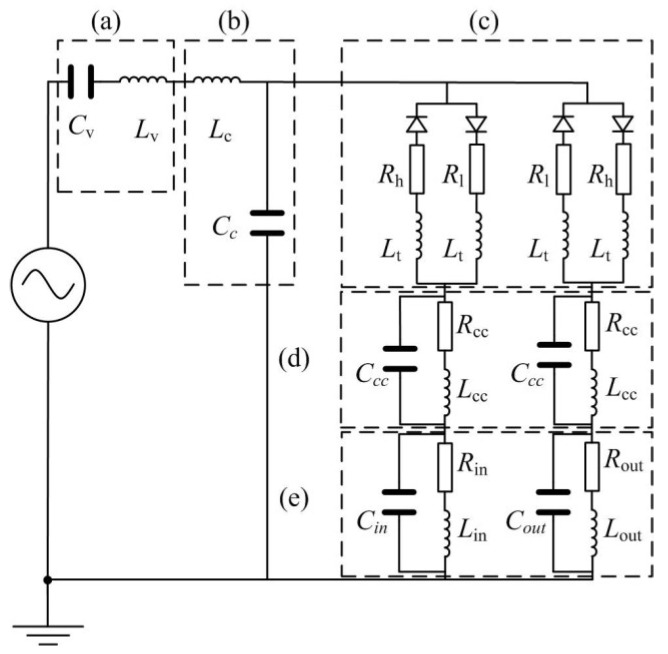
Equivalent circuit diagram of valveless piezoelectric pump with multistage Y-shape treelike bifurcate tubes: (**a**) Piezoelectric vibrator; (**b**) Pump chamber; (**c**) Multistage Y-shape treelike bifurcate tube; (**d**) Collection chamber; (**e**) Inlet/Outlet tube.

Based on the analogy results of fluid and electrical models of parts of the valveless piezoelectric pump mentioned above, as well as the mathematical model of the valveless piezoelectric pump, the equivalent circuit diagram of the valveless piezoelectric pump is obtained, as shown in Figure 6. The subscripts ‘in’ and ‘out’ represent the inlet pipe and outlet pipe of the valveless piezoelectric pump, respectively. As can be seen from Figure 1, the piezoelectric pump consists of a piezoelectric vibrator, circular pump chamber, multistage Y-shape treelike bifurcate tubes, square collection chamber and inlet/outlet tubes. According to the working principle of the piezoelectric pump, when the vibrator uplifts outward, the fluid flows into the chamber through the inlet/outlet tubes, collection chambers and Y-shape treelike bifurcate tubes. This process can be equivalent to the input voltage of the circuit model in the positive half cycle, and the current flows through the above equivalent elements respectively. When the vibrator depresses inward, the fluid flows out of the chamber through the tubes, collection chambers and the inlet/outlet tubes. This process can be equivalent to the input voltage of the circuit model in the negative half cycle, and the current flows through each equivalent component.

**Figure 7:**
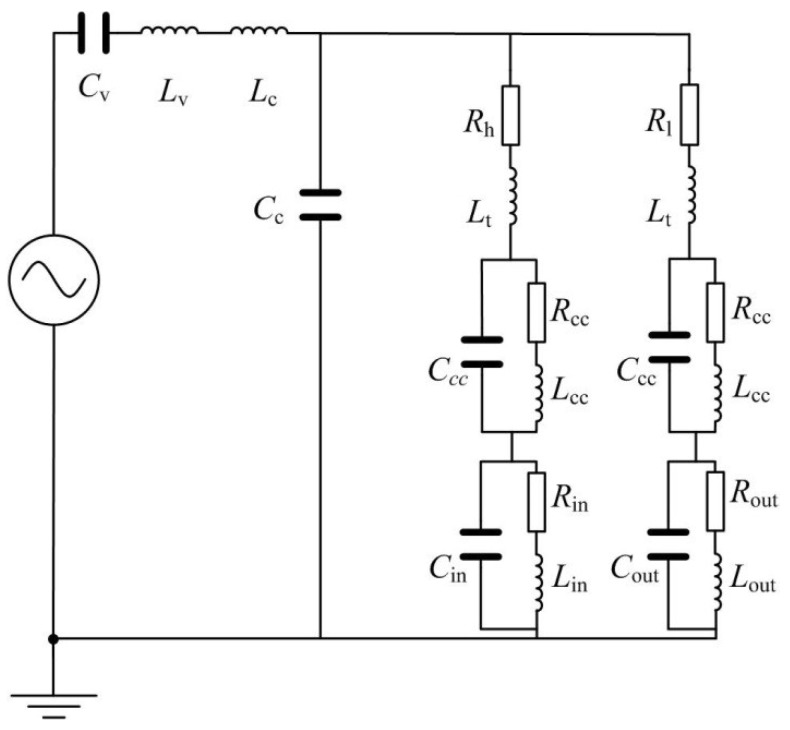
Equivalent circuit diagram when the input voltage is in the negative half-period.

It can be concluded from the circuit diagram that when the input voltage is in the positive half-period, the existence of conduction and diode blocking results in a higher electric resistance for the branch circuits at the outlet section compared with the inlet section, which creates a larger branch current at the inlet section compared to the outlet section. Therefore, this half period can be treated as the suction period of the valveless piezoelectric pump. Similarly, when the input voltage is in the negative half-period, the existence of conduction and blocking of diode results in a higher electric resistance for the branch circuits at the inlet section compared with the boutlet section, which makes a larger branch current at the outlet section compared to the inlet section. Therefore, this half period can be treated as the discharge period of the valveless piezoelectric pump. At this time, the current flowing through *R*_out_, *L*_out_ and *C*_out_ can be equivalent to the output flow of the piezoelectric pump. According to the unidirectional conduction characteristics of the diode, when the input voltage is in the negative half-period, and the corresponding equivalent circuit model is shown in Figure 7.

A commercial EDA software package (Multisim 12.0, National Instruments, Austin, TX, USA) is adopted for the simulation of the equivalent circuit of the valveless piezoelectric pump. The results are compared with experimental pumping flow results to demonstrate the feasibility and validity of the proposed equivalent circuit.

## 5. Results and Discussion

By substituting the related parameter values in Table 1 into the equations, the values of the circuit components of the valveless piezoelectric pump with multistage Y-shape treelike bifurcate tubes are obtained. However, the loss coefficient for dividing flow and merging flow of multistage Y-shape treelike bifurcate tubes are different, therefore, simulation for multistage Y-shape treelike bifurcate tubes with ANSYS CFX is carried out in this research. 

**Figure 8:**
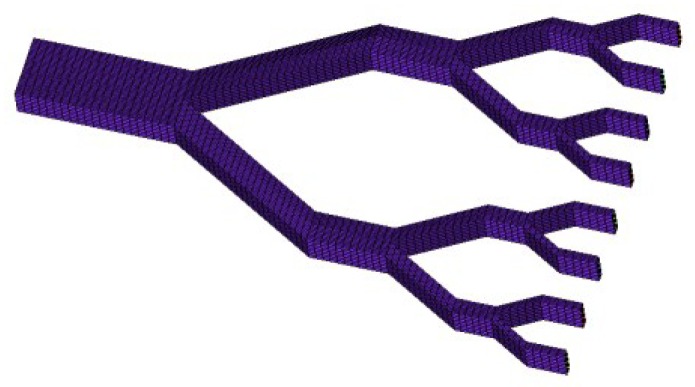
Finite element model of the tube.

**Figure 9:**
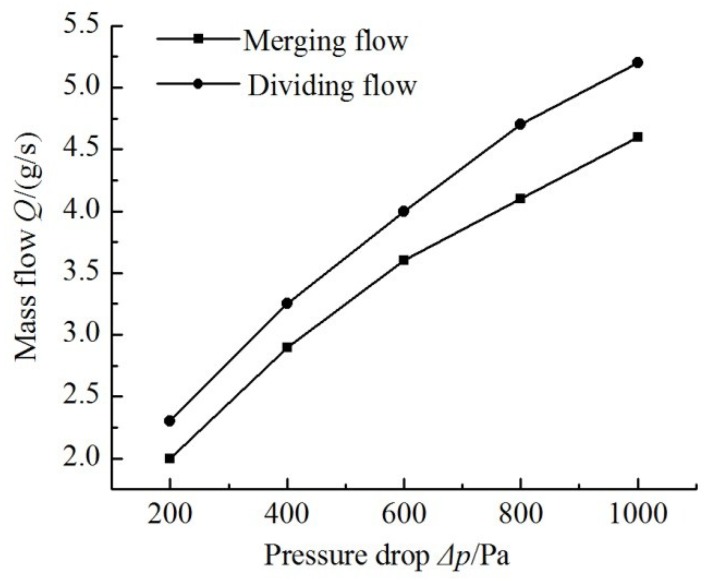
Curves of mass flow versus pressure drop.

The finite-element model of flow field of multistage Y-shape treelike bifurcate tubes is shown in Figure 8. By importing this model into ANSYS CFX for flow field simulation, the relation curve of pressure difference between the two ends of tubes and outlet flow in positive flowing and negative flowing is obtained, as shown in Figure 9. 

It can be seen from Figure 9 that the relation curve of mass flow and pressure drops is similar to a straight line. Therefore, according to Equation (1), the flow resistance of the multistage Y-shape treelike bifurcate tube is the cotangent function of angle of the “straight line” in Figure 9. The equivalent circuit parameters have been listed in Table 2. 

**Figure 10:**
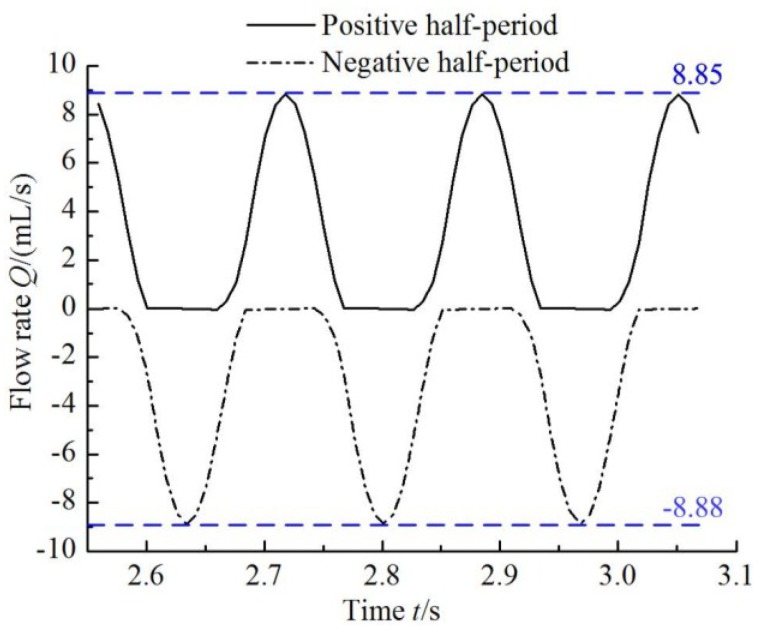
Transient flow rate curves of branches of outlet tube.

The transient flow rate curves of the outlet tube branches in the simulation of the equivalent circuit of the valveless piezoelectric pump are shown in Figure 10. 

From Figure 10 it can be seen that the output flow rate in negative half-period is larger than that in the positive half-period, therefore, the net flow of the valveless piezoelectric pump with multistage Y-shape treelike bifurcate tubes can be obtained by subtracting the integration values of the two current functions. 

**Figure 11:**
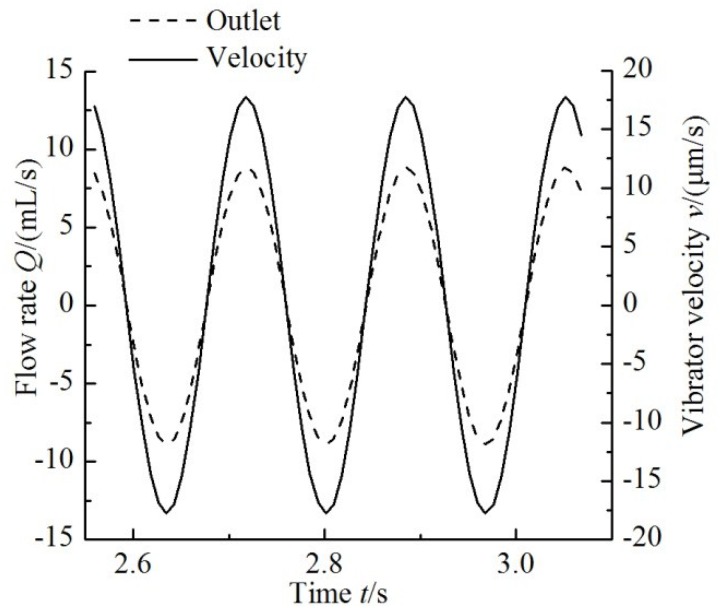
Curves of flow rate and vibrator velocity.

Figure 11 shows the variation of the flow rate with the change in time at the outlet and the velocity of the piezoelectric vibrator for a driving frequency of 6 Hz. The flow rate curve of the pump and the velocity curve of the vibrator are in the same phase. 

**Figure 12:**
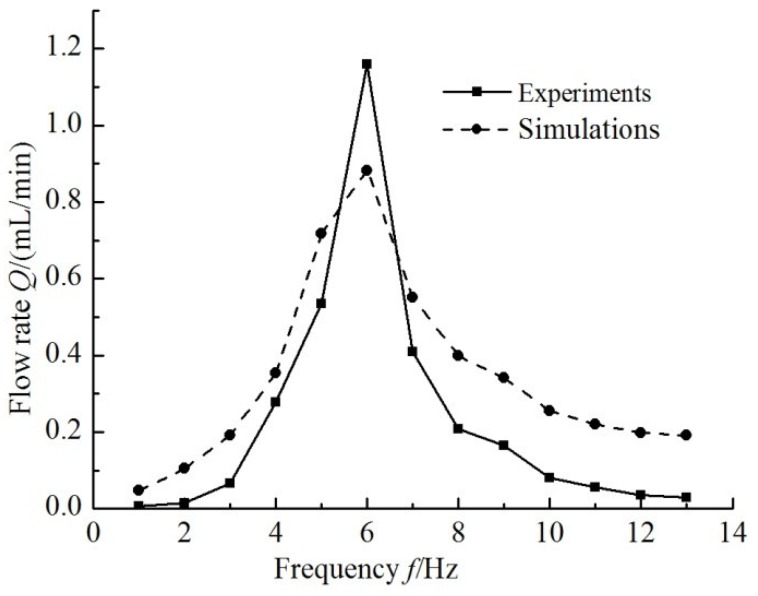
Comparison of experimental flow rate with simulation flow rate.

By changing the drive frequency of equivalent circuit of the valveless piezoelectric pump with multistage Y-shape treelike bifurcate tubes, the output current of outlet tube is obtained. Then its integrated values are the outflow of the piezoelectric pump. The experimental and theoretical curves of outflow of the pump that changes with frequency are shown in Figure 12. 

The experimental maximum flow rate of the valveless piezoelectric pump is 1.16 mL/min under 100 V (6 Hz) power supply. The theoretical maximum flow rate of the pump is 0.88 mL/min, while the largest relative error (85.71%) between the theoretical calculation results and experimental results of the pump appear at the frequency of 2 Hz. This deviation is due to the longer multistage Y-shape treelike bifurcate tubes and larger volume; while the fluid capacitance of the tube is neglected in the equivalent circuit model, resulting in a relatively large deviation between theoretical and experimental outflow results at low frequency. 

**Figure 13:**
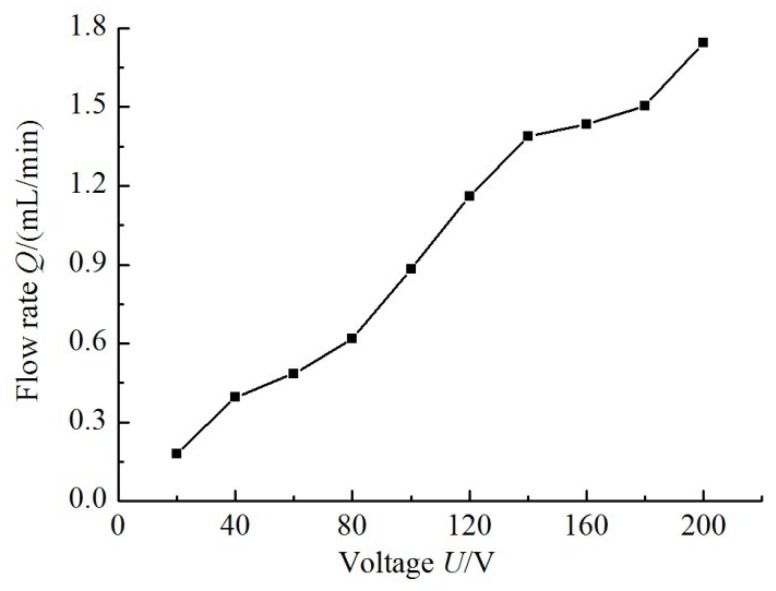
Simulation flow rate with various driving voltage inputs at 6 Hz.

Figure 13 shows the relation curve between the simulation flow rate and the input voltage when the frequency is 6 Hz. It is seen that the flow rate is positively related to the driving voltage.

Using the Laplace transform analysis method to analyze the circuit shown in Figure 7, the system transfer function of the output of the equivalent model can be obtained:

(16) $$\begin{array}{ll} H(s) & =[(R_{out}+sL_{out})sC_{out}+1]\{[\frac{2R_{d}+2R_{e}+R_{h}+2sL_{t}+R_{l}}{R_{d}+R_{e}+R_{l}+sL_{t}}\\ & +(R_{d}+R_{e}+R_{h}+sL_{t})sC_{c}][\frac{1}{sC_{v}}+s(L_{v}+L_{c})]+1\} \end{array}$$

(17) $$R_{d}=(R_{cc}+sL_{cc})∥\frac{1}{sC_{cc}}$$

(18) $$R_{e}=(R_{in}+sL_{in})∥\frac{1}{sC_{in}}=(R_{out}+sL_{out})∥\frac{1}{sC_{out}}$$

Because of the higher order of the circuit system, the manual calculation is too complicated. The simulation software Multisim 12.0 is used to calculate the pole-zero of the equation, the poles of the system are located in the left half plane of the complex plane, that is, the system has good robustness.

## 6. Conclusions

In this paper, based on the liquid-electric analogy theory, the equivalent circuit model of a valveless piezoelectric pump with multistage Y-shape treelike bifurcate tubes is developed. Based on the structural dimensions and material parameters of the piezoelectric pump, the parameters for circuit components of equivalent circuit of the valveless piezoelectric pump are determined, and the flow resistance of the tube is obtained by finite element software calculations; the simulation results show that at the driving frequency of 6 Hz, the theoretical maximum flow rate is 0.88 mL/min. The electrical model is verified by experimental results. The experimental maximum flow rate of the valveless pump is 1.16 mL/min under 100 V (6 Hz) power supply. The largest relative error between theoretical calculation results and experimental results of the pump is observed at the frequency of 2 Hz, and calculated to be 85.71%. The above results show that the circuit model can predict the output performance of valveless piezoelectric pumps quickly and effectively.

## Figures and Tables

**Table 1:** Parameters of the valveless piezoelectric pump with multistage Y-shape treelike bifurcate tubes.

Parameter	Symbol	Value
Diameter of pump chamber (mm)	*d*	40
Depth of pump chamber (mm)	*h*	2
Width of mother tube (mm)	*a*	4
Length of mother tube (mm)	*l*	10
Bifurcation angle of tubes (°)	2*α*	60
Density of PZT (kg/m^3^)	*ρ*_PZT_	7800
Radius of PZT (mm)	*r*_PZT_	15
Thickness of PZT (mm)	*h*_PZT_	0.2
Radius of metal substrate (mm)	*r*_membrane_	50
Thickness of metal substrate (mm)	*h*_membrane_	0.2
Elastic modulus of metal substrate (GPa)	*E*_membrane_	120
Density of metal substrate (kg/m^3^)	*ρ*_membrane_	7850
Poisson ratio of metal substrate	*ν*_membrane_	0.33

**Table 2:** Calculation results for equivalent circuit components.

Component Name	Symbol	Value
Vibrator capacitance	*C*_v_	37.87 pF
Vibrator inductance	*L*_v_	4371.135 H
Chamber inductance	*L*_c_	1592.4 H
Chamber capacitance	*C*_c_	1.1418 fF
Y-shape tube resistance (high resistance state)	*R*_h_	242 MΩ
Y-shape tube resistance (Low resistance state)	*R*_l_	240 MΩ
Y-shape tube inductance	*L*_t_	4.6875 MH
Collection chamber inductance	*L*_cc_	0.2 MH
Collection chamber capacitance	*C*_cc_	0.15 fF
Collection chamber resistance	*R*_cc_	1.8577 MΩ
Inlet/Outlet tube inductance	*L*_in_ */L*_out_	37.689 MH
Inlet/Outlet tube capacitance	*C*_in_ */C*_out_	0.4824 fF
Inlet/Outlet tube resistance	*R*_in_ */R*_out_	178.4 MΩ
